# Investigation of *Aspergillus fumigatus* biofilm formation by various “omics” approaches

**DOI:** 10.3389/fmicb.2013.00013

**Published:** 2013-02-12

**Authors:** Laetitia Muszkieta, Anne Beauvais, Vera Pähtz, John G. Gibbons, Véronique Anton Leberre, Rémi Beau, Kazutoshi Shibuya, Antonis Rokas, Jean M. Francois, Olaf Kniemeyer, Axel A. Brakhage, Jean P. Latgé

**Affiliations:** ^1^Unité des Aspergillus, Institut PasteurParis Cedex, France; ^2^Department of Molecular and Applied Microbiology, Leibniz Institute for Natural Product Research Infection Biology - Hans Knöll Institute and Department of Microbiology and Molecular Biology, Institute of Microbiology, Friedrich Schiller University JenaJena, Germany; ^3^Integrated Research and Treatment Center, Center for Sepsis Control and Care Jena, University HospitalJena, Germany; ^4^Department of Biological Sciences, Vanderbilt UniversityNashville, TN, USA; ^5^Université de Toulouse; INSA, UPS, INP; LISBPToulouse, France; ^6^INRA, UMR792, Ingénierie des Systèmes Biologiques et des ProcédésToulouse, France; ^7^CNRS, UMR5504Toulouse, France; ^8^Department of Pathology, Omori Hospital, Toho University School of MedicineOta-Ku, Tokyo, Japan

**Keywords:** biofilm, transcriptomic, proteomic analysis, RNA-sequencing, RNA-seq, *Aspergillus fumigatus*

## Abstract

In the lung, *Aspergillus fumigatus* usually forms a dense colony of filaments embedded in a polymeric extracellular matrix called biofilm (BF). This extracellular matrix embeds and glues hyphae together and protects the fungus from an outside hostile environment. This extracellular matrix is absent in fungal colonies grown under classical liquid shake conditions (PL), which were historically used to understand *A. fumigatus* pathobiology. Recent works have shown that the fungus in this aerial grown BF-like state exhibits reduced susceptibility to antifungal drugs and undergoes major metabolic changes that are thought to be associated to virulence. These differences in pathological and physiological characteristics between BF and liquid shake conditions suggest that the PL condition is a poor *in vitro* disease model. In the laboratory, *A. fumigatus* mycelium embedded by the extracellular matrix can be produced *in vitro* in aerial condition using an agar-based medium. To provide a global and accurate understanding of *A. fumigatus in vitro* BF growth, we utilized microarray, RNA-sequencing, and proteomic analysis to compare the global gene and protein expression profiles of *A. fumigatus* grown under BF and PL conditions. In this review, we will present the different signatures obtained with these three “omics” methods. We will discuss the advantages and limitations of each method and their complementarity.

## Introduction

**Figure 1:**
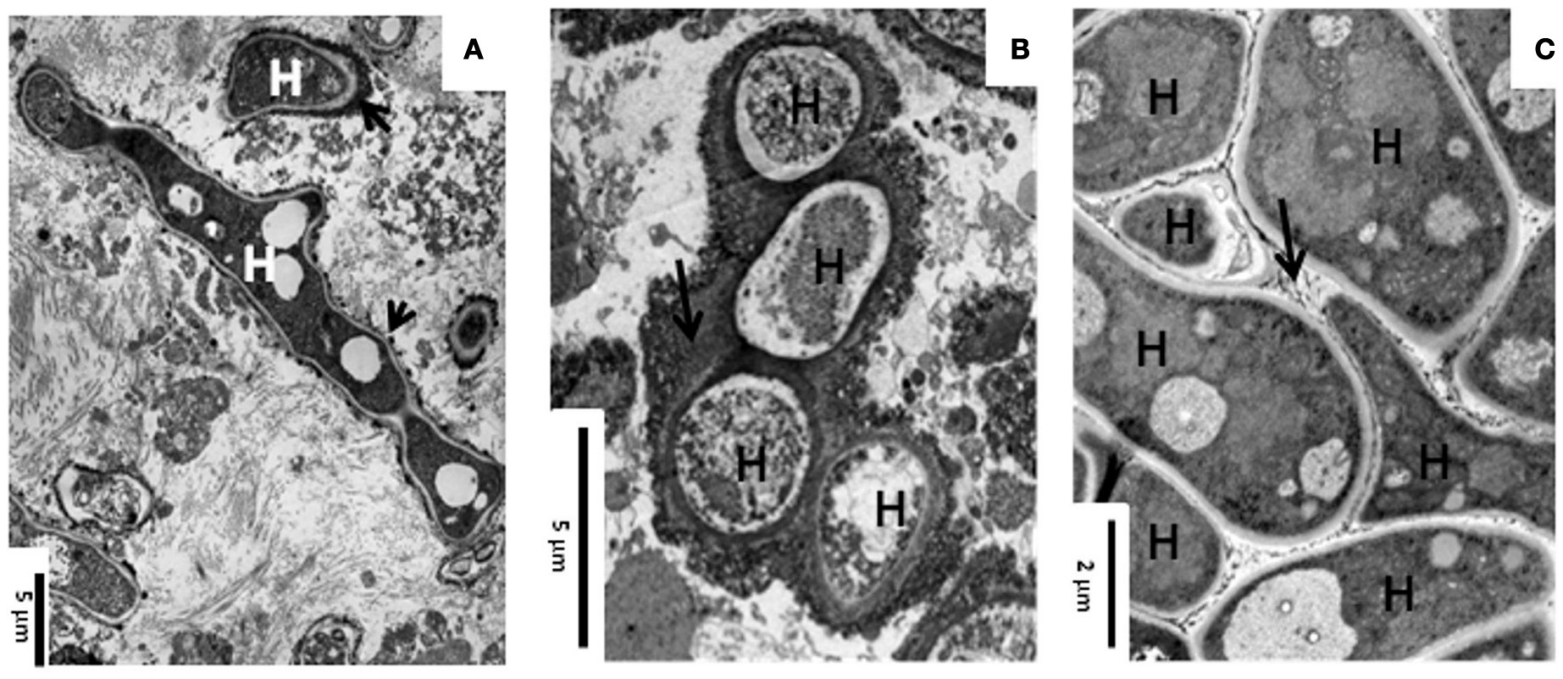
**Transmission electron microscopy showing the ultrastructure of *A. fumigatus* biofilm *in vivo* and *in vitro*. (A)** Invasive aspergillosis in human lung; **(B)** Aspergilloma in human lung; and **(C)** 24 h static and aerial culture of *A. fumigatus* at 30°C. Note the presence of an extracellular material (ECM, arrow) at the surface of the hyphae (H).

During lung infection, *Aspergillus fumigatus* hyphae are covered by an extracellular matrix (Figures 1A,B) (Loussert et al., 2010). In the case of aspergilloma, hyphae are embedded together in this dense extracellular matrix whereas in invasive aspergillosis hyphae are individually engulfed in the matrix (Figures 1A,B) (Beauvais et al., 2007; Muller et al., 2011). This extracellular matrix protects the fungus against host defense reactions as well as antifungal drugs. The *in vivo* composition of the mycelial extracellular matrix of *A. fumigatus* has been reported during host infection (Loussert et al., 2010). The extracellular matrix is composed of polysaccharides, pigment, and proteins. *A. fumigatus* biofilm (BF) condition can be reproduced *in vitro*. Indeed, the mycelium growing on porous plastic film deposited on the surface of agar medium plate is able to form an extracellular matrix with a composition closely similar to the *in vivo* with tightly bound hyphae (Figure 1C) (Beauvais et al., 2007). In contrast, this extracellular matrix is absent in mycelia grown in shake cultures and hyphae are only loosely associated. These differences in organizational and physiological characteristics between the mycelium growing under “planktonic” or “biofilm” condition are associated with specific transcriptional and translational signatures. As the development of the fungal BF *in vivo* is more close to aerial colony grown on a solid substratum *in vitro*, it is expected that an analysis of the colony physiology may help to understand the *in vivo* growth of *A. fumigatus* in patients.

High-throughput technologies enable quantitative monitoring of the abundance of various biological molecules and allow quantification of their variation between two different conditions on a genomic scale. Omics approaches involve high-throughput technologies that enable the measurement of global changes in the abundance of mRNA transcripts (transcriptomic), proteins (proteomic), and other biomolecular components (metabolomic) in complex biological systems as a result of chemical perturbation or transition of developmental stages (Nie et al., 2007; Hawkins et al., 2010; Ozsolak and Milos, 2011). Using “omics” methods to compare the mycelium obtained in aerial condition vs. the mycelium growing in submerged condition may allow us to identify the biological process important during the BF growth.

In this review, we will present the different transcriptional and translational signature obtained by using transcriptomic (microarray and RNA-sequencing) and proteomic analyses of BF grown mycelium in comparison to submerged mycelium. In addition, since the application of omics technologies is quite at its infancy in the *A. fumigatus* field, comparison of these three “omics” methods makes it possible to highlight the advantages and limitations or complementarity of these methods.

## Transcriptomic analysis

*A. fumigatus* ATCC_46645 was the wild-type strain used in these analyses. This genome is composed of 9.926 predicted genes organized in eight chromosomes for a total size of 29.4 Mb (Niermann et al., 2005). Total RNA of aerial colony or submerged mycelium were obtained as described previously (Gibbons et al., 2012).

### Data obtained with microarrays and RNA-sequencing analysis

Four biological replicates of the microarray experiment were performed, each time with a reciprocal labeling protocol (“dye-swap”), which served both as a labeling control and technical replicate. The microarrays analysis was realized by using the AF gene chip microarrays that cover about 9600 Open Reading Frames from genome of strain ATCC_46645, sequenced by J. Craig Venter Institute (JCVI), The Institute for Genomic Research (TIGR). Scanning was performed with an Axon scanner 4000A and the resulting images were analyzed by using GenePix Pro 6.01 software. The Bioplot software was used for statistical analysis. Quantile normalization was applied to the whole data set to account for variation between slides. Expression ratio cutoff of 2.0 and 0.5 were applied to select differentially expressed genes with a *p*-value <0.05 (Student's *t*-test). 359 genes differentially expressed in the BF condition as compared to submerged condition were identified. Among them, 193 and 169 genes were up or down regulated, respectively, under the BF growth conditions. The differentially expressed genes were classified according to the functional catalog FunCat. 66.84 and 59.17% of the up and down regulated genes were functionally annotated, which led to the identification of 6 functional categories significantly up regulated and 14 functional categories down regulated in *A. fumigatus* BF (*p* < 0.05 Fisher's Exact Test) (Table 1). However, when we considered the percentage of genes up or down regulated per category, this percentage was too low to ascertain the global up or down regulation of any of these functional categories.

**Table 1:** **Functional categorization of the differentially expressed genes in the biofilm condition by using microarrays**.

	**Number of hits**	**Total of hits in the category**	**% hits of the category**	**Fisher's exact test *p*-value**
**CATEGORIES UP REGULATED IN BIOFILM BY USING MICROARRAYS**				
Translation	27	214	12.6	1.05E-13
Ribosome biogenesis	25	255	9.8	2.75E-10
Fungal/microorganismic cell type differentiation	21	485	4.3	0.0034
Lipid, fatty acid, and isoprenoid metabolism	8	746	1.1	0.0168
Protein binding	45	867	5.2	0.0337
RNA synthesis	11	1511	0.7	0.0341
**CATEGORIES DOWN REGULATED IN BIOFILM BY USING MICROARRAYS**				
Disease, virulence, and defense	21	379	5.5	1.66E-06
Detoxification	20	407	4.9	1.89E-05
Respiration	11	155	7.1	7.34E-05
Fermentation	8	91	8.8	0.0002
DNA processing	1	578	0.2	0.0006
Protein binding	15	1511	1.0	0.0082
Nucleic acid binding	5	754	0.7	0.0108
Nucleotide/nucleoside/nucleobase metabolism	1	371	0.3	0.0213
Metabolism of vitamins, cofactors, and prosthetic groups	11	320	3.4	0.0265
Cell cycle	5	690	0.7	0.0287
Complex cofactor/cosubstrate/vitamine binding	14	441	3.2	0.0343
Stress response	18	635	2.8	0.0357
Secondary metabolism	16	551	2.9	0.0389
Lipid, fatty acid, and isoprenoid metabolism	20	746	2.7	0.0499

The analysis of the transcriptional signature of the *A. fumigatus* BF grown under the same conditions was already published by Gibbons et al. (2012) by using RNA-sequencing. This method identified 10-fold more genes differentially expressed in the BF than microarrays. Among the 3729 differentially expressed genes, 2564 genes were up regulated and 1164 genes were down regulated in the BF. The functional categorization of the differentially expressed genes showed a total of 31 up regulated and 31 down regulated functional categories under BF growth conditions (Tables 2, 3). Among the different categories identified, 5 of the 6 up regulated categories and 9 of 14 down regulated categories of the microarrays analysis are retrieved, respectively, among up regulated and down regulated categories identified by using RNA-sequencing (Tables 1, 2, 3). Among the most highly enriched categories of the RNA-sequencing data, the categories linked to transport, detoxification, disease, virulence and defense, and homeostasis were significantly up regulated whereas the categories linked to carbohydrate metabolism such as glycolysis/glucogenesis and tricarboxylic-acid cycle were significantly down regulated.

**Table 2:** **Functional categorization of the up regulated biofilm genes obtained by using RNA-sequencing**.

**Categories up regulated in RNA-sequencing**	**Number of hits**	**Total of hits in the category**	**% hits of the category**	**Fisher's exact test *p*-value**
Transport facilities	286	660	43.3	2.40E-19
Transported compounds (substrates)	476	1328	35.8	7.33E-13
Regulation of protein activity	83	499	16.6	7.99E-10
Cell cycle	128	690	18.6	1.52E-09
DNA processing	103	578	17.8	2.86E-09
Nucleus	43	304	14.1	5.55E-09
Nucleic acid binding	153	754	20.3	2.59E-07
Cellular signaling	89	485	18.4	3.50E-07
Ribosome biogenesis	108	255	42.4	4.73E-07
Homeostasis	118	286	41.3	6.88E-07
Protein binding	349	867	40.3	7.35E-07
RNA synthesis	184	1511	12.2	9.77E-07
Detoxification	154	407	37.8	7.32E-06
Regulation by	45	263	17.1	3.11E-05
Nucleotide/nucleoside/nucleobase binding	178	796	22.4	0.0001
Cytoskeleton/structural proteins	50	276	18.1	0.0001
Transport routes	352	1094	32.2	0.0006
Lipid, fatty acid, and isoprenoid metabolism	247	746	33.1	0.0010
Aminoacyl-tRNA-synthetases	3	44	6.8	0.0010
Mitochondrion	25	151	16.6	0.0012
Bud/growth tip	1	29	3.4	0.0014
Translation	81	214	37.9	0.0014
Stress response	145	635	22.8	0.0023
Cell growth/morphogenesis	74	348	21.3	0.0037
Transmembrane signal transduction	34	171	19.9	0.0155
RNA processing	98	425	23.1	0.0184
Glycolysis and gluconeogenesis	21	115	18.3	0.0205
Disease, virulence, and defense	126	379	33.2	0.0208
Phosphate metabolism	139	575	24.2	0.0350
Structural protein binding	9	58	15.5	0.0385
Metabolism of vitamins, cofactors, and prosthetic groups	74	320	23.1	0.0470

**Table 3:** **Functional categorization of the down regulated biofilm genes obtained by RNA-sequencing**.

**Categories down regulated in RNA-sequencing**	**Number of hits**	**Total of hits in the category**	**% hits of the category**	**Fisher's exact test *p*-value**
Transport facilities	32	660	4.8	6.81E-12
Ribosome biogenesis	6	255	2.4	8.17E-09
Glycolysis and gluconeogenesis	37	115	32.2	1.58E-08
Transported compounds (substrates)	116	1328	8.7	2.35E-06
Nucleus	62	304	20.4	4.84E-05
Stress response	111	635	17.5	7.11E-05
RNA synthesis	140	1511	9.3	0.0004
Regulation of protein activity	87	499	17.4	0.0006
Disease, virulence, and defense	28	379	7.4	0.0016
Bud/growth tip	10	29	34.5	0.0018
Fermentation	22	91	24.2	0.0018
Complex cofactor/cosubstrate/vitamine binding	76	441	17.2	0.0020
C-compound and carbohydrate metabolism	200	1346	14.9	0.0021
Tricarboxylic-acid pathway (citrate cycle, Krebs cycle, and TCA cycle)	15	53	28.3	0.0023
Translation	13	214	6.1	0.0029
Transport routes	107	1094	9.8	0.0036
Protein folding and stabilization	28	132	21.2	0.0045
Nucleic acid binding	116	754	15.4	0.0090
Transmembrane signal transduction	33	171	19.3	0.0090
DNA processing	92	578	15.9	0.0092
Fungal/microorganismic cell type differentiation	79	485	16.3	0.0093
Cell growth/morphogenesis	59	348	17.0	0.0115
Extracellular metabolism	1	56	1.8	0.0122
Nitrogen, sulfur and selenium metabolism	47	275	17.1	0.0188
Homeostasis	23	286	8.0	0.0210
Nucleotide/nucleoside/nucleobase metabolism	32	371	8.6	0.0223
Respiration	28	155	18.1	0.0352
Cell cycle	103	690	14.9	0.0359
Metabolism of energy reserves (e.g., glycogen and trehalose)	14	66	21.2	0.0373
Anaplerotic reactions	2	3	66.7	0.0422
Nucleotide/nucleoside/nucleobase binding	116	796	14.6	0.0485

### Transcriptomic signature: microarray vs. RNA-sequencing analysis

Whereas the microarray analysis leads to the identification of hundreds of differentially expressed genes, RNA-sequencing allowed the identification of thousands of genes, which were differentially expressed in the BF. For several categories more than 30% of hits constituting a specific FunCat category were differentially expressed in the RNA-sequencing experiment. In constrast, in microarray analyses no more than 12% of the hits belonging to one category were differentially expressed (Tables 2, 3). Thus, RNA-sequencing allows a more robust identification of functional categories that represent the transcriptional signature of the BF growth of *A. fumigatus* (Tables 2, 3). Several reasons could explain the difference between signatures obtained with these two methods and justify the current replacement of microarrays analysis by RNA-sequencing data.

The development of microarrays enabled for the first time the simultaneous analysis of the expression levels of thousands of known or putative transcripts. However, microarrays provide mRNA expression pattern data based on the high-throughput and semi quantitative analysis of fluorescence signaling intensities (Morozova et al., 2009). However, this technique has limitations. As the technique relies on hybridization, it poses a range of potential problems such as interfering background hybridization levels, cross hybridization, difference in probe hybridization properties, and dye binding variances. This technological bias means that microarrays do not quantify easily and properly the expression pattern of low abundant transcripts since low intensity fluorescence signals are difficult to distinguish numerically and statistically from the background noise (Roy et al., 2011). Conversely, signal saturation can occur at high intensities and limits the ability to compare transcripts that are expressed at very high levels. In comparison, RNA-sequencing offers several major advantages. Firstly, RNA-sequencing allows quantifying gene expression levels precisely without any background by sequencing each transcript independently (Wang et al., 2009). Secondly, RNA-sequencing is very sensitive and can detect a larger dynamic range of gene expression levels in comparison to microarrays, without a lack of sensitivity for genes expressed at very low or very high levels. Furthermore, RNA-sequencing has showed a better reproducibility for both technical and biological replicates. These methodological and technical variations inherent to the methodologies themselves can explain the difference in the number of differentially expressed genes obtained by applying two methods to one experimental set-up.

**Figure 2:**
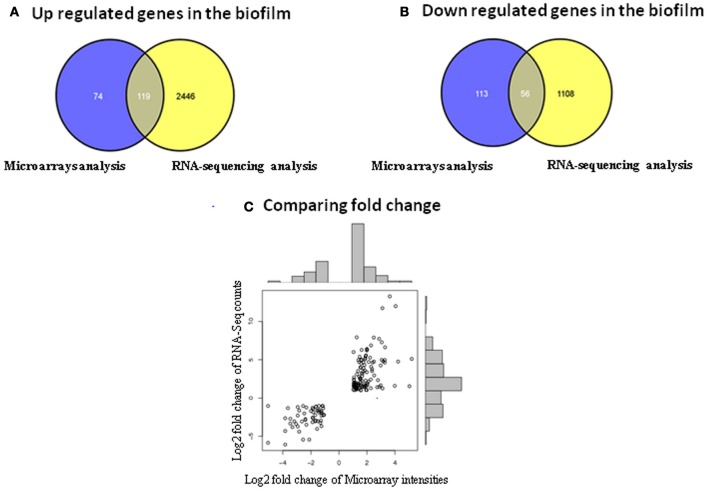
**Identification of the differentially expressed genes common to both transcriptomic methods. (A)** Comparison of the up regulated genes obtained with microarray and RNA-sequencing analysis in the biofilm. **(B)** Comparison of the down regulated genes obtained with microarray and RNA-sequencing analysis in the biofilm. **(C)** Comparison of the fold change obtained with microarray and RNA-sequencing analysis.

In spite of these discrepancies, it was observed that among the 193 up regulated genes identified by microarrays, 119 were also up regulated in the RNA-sequencing data (Figure 2A). Among the 169 down regulated genes identified in the microarrays only 56 were shown to be down regulated in the RNA-sequencing analysis (Figure 2B). Thus, ~49% of the differentially expressed genes identified with microarrays were also retrieved in the RNA-sequencing data with a positive correlation of *p* = 0.82 (Pearson correlation) (Figure 2C). Some of the common differentially expressed genes found in both transcriptomic methods are discussed below (Table 4).

**Table 4:** **List of differentially expressed genes common to both transcriptomic methods used**.

**Accession number**	**Gene function**	**Microarray ratios of intensities**	**Log2 fold change of ratio intensities**	**RNA-seq ratio of counts**	**Log2 fold change of counts**
AFUA_8G00200	CalO6, putative	12.39	3.63	9885.95	13.27
AFUA_1G17250	Conidial hydrophobin RodB	16.54	4.05	4118.35	12.01
AFUA_6G11850	Hypothetical protein	8.61	3.11	3408.58	11.73
AFUA_7G06620	Related to L-fucose permease, putative	2.41	1.27	243.13	7.93
AFUA_4G03240	Cell wall galactomannoprotein Mp1	5.6	2.49	238.51	7.90
AFUA_8G00900	Cell surface antigen spherulin 4, putative	7.31	2.87	212.52	7.73
AFUA_5G08800	Hypothetical protein	8.32	3.06	155.28	7.28
AFUA_3G01500	Hypothetical protein	4.89	2.29	117.44	6.88
AFUA_5G13250	DUF614 domain protein	9.8	3.29	98.50	6.62
AFUA_1G14560	Alpha-mannosidase	3.99	2.00	84.72	6.40
AFUA_1G00990	Short chain dehydrogenase/reductase family protein	4.02	2.01	79.69	6.32
AFUA_4G13050	Hypothetical protein	3.07	1.62	77.73	6.28
AFUA_4G01350	Hypothetical protein	3.97	1.99	73.93	6.21
AFUA_1G10390	ABC multidrug transporter, putative	2.06	1.04	64.23	6.01
AFUA_4G03330	DUF590 domain protein, putative	3.83	1.94	47.61	5.57
AFUA_1G03350	Alpha-1,3-glucanase, putative	3.73	1.90	44.20	5.47
AFUA_7G00420	Hypothetical protein	2.79	1.48	41.86	5.39
AFUA_4G07810	L-serine dehydratase, putative	4.67	2.22	38.63	5.27
AFUA_4G08370	Conserved hypothetical protein	3.68	1.88	37.09	5.21
AFUA_5G02330	Major allergen Asp F1	36.71	5.20	34.45	5.11
AFUA_6G02220	MFS toxin efflux pump, putative	3.64	1.86	33.48	5.07
AFUA_8G00410	Methionine aminopeptidase, type II, putative	3.02	1.59	32.34	5.02
AFUA_6G14340	Related to berberine bridge enzyme [imported]	8.28	3.05	31.45	4.98
AFUA_1G12560	Endo-1,4-beta-glucanase, putative	9.66	3.27	31.32	4.97
AFUA_1G14800	Hypothetical protein	2.62	1.39	30.98	4.95
AFUA_2G15200	Conserved hypothetical protein	3.04	1.60	30.59	4.94
AFUA_4G08380	Hypothetical protein	8.98	3.17	27.58	4.79
AFUA_2G15290	DUF636 domain protein	18.7	4.22	27.06	4.76
AFUA_2G09030	Secreted dipeptidyl peptidase	5.67	2.50	27.02	4.76
AFUA_3G03670	ABC multidrug transporter, putative	4.38	2.13	26.18	4.71
AFUA_5G13950	Conserved hypothetical protein	2.72	1.44	25.98	4.70
AFUA_1G02290	Conserved hypothetical protein	5.28	2.40	25.11	4.65
AFUA_1G06350	Virulence related protein (Cap20), putative	3.35	1.74	25.04	4.65
AFUA_1G14430	Chitin binding protein, putative	9.66	3.27	24.90	4.64
AFUA_4G09400	Aspartic-type endopeptidase (AP3), putative	3	1.58	23.10	4.53
AFUA_2G00500	Conserved hypothetical protein	3.54	1.82	19.95	4.32
AFUA_3G03650	Acetyltransferase, GNAT family, putative	7.88	2.98	19.03	4.25
AFUA_3G08610	DUF124 domain protein	4.91	2.30	17.08	4.09
AFUA_6G13830	Oxidoreductase, short chain dehydrogenase/reductase family	2.89	1.53	16.14	4.01
AFUA_1G00930	Hypothetical protein	2.95	1.56	15.40	3.94
AFUA_4G09390	Conserved hypothetical protein	3.38	1.76	15.15	3.92
AFUA_3G03640	Siderochrome-iron transporter (MirB), putative	5.17	2.37	13.62	3.77
AFUA_6G12170	FKBP-type peptidyl-prolyl isomerase, putative	3.61	1.85	11.97	3.58
AFUA_7G00250	Tubulin beta-2 subunit	5.13	2.36	11.46	3.52
AFUA_3G02010	Cytochrome P450 monooxygenase, putative	2.57	1.36	11.46	3.52
AFUA_8G06490	Conserved hypothetical protein	3.3	1.72	11.15	3.48
AFUA_5G03780	L-PSP endoribonuclease family protein	3.96	1.99	10.34	3.37
AFUA_2G13500	Hypothetical protein	3.05	1.61	9.47	3.24
AFUA_1G01160	Salivary apyrase, putative	3.22	1.69	9.15	3.19
AFUA_2G16060	Conserved hypothetical protein	2.43	1.28	8.56	3.10
AFUA_2G04080	GPR/FUN34 family protein	5.72	2.52	7.70	2.94
AFUA_7G06540	Low-specificity L-threonine aldolase, putative	3.15	1.66	7.19	2.85
AFUA_4G09220	Hypothetical protein	2.72	1.44	6.85	2.78
AFUA_8G02060	Glycan biosynthesis protein (PiGL), putative	2.11	1.08	6.47	2.69
AFUA_3G00340	Glycosyl hydrolase, putative	2.86	1.52	6.11	2.61
AFUA_3G12300	Ribosomal L22e protein family	2.82	1.50	5.60	2.49
AFUA_4G00200	F-box domain protein	2.91	1.54	5.45	2.45
AFUA_2G16070	Urease accessory protein UreD	4.84	2.28	5.34	2.42
AFUA_5G06320	Membrane biogenesis protein (Yop1), putative	2.1	1.07	5.30	2.41
AFUA_2G15130	ABC multidrug transporter, putative	6.9	2.79	5.03	2.33
AFUA_1G03110	Ribosomal protein L29	2.1	1.07	4.98	2.31
AFUA_5G14930	Conserved hypothetical protein	3.74	1.90	4.87	2.29
AFUA_6G12660	40s ribosomal protein	3.09	1.63	4.74	2.24
AFUA_8G00960	Cytochrome P450, putative	4.29	2.10	4.43	2.15
AFUA_1G15020	40s ribosomal protein S5	2.88	1.53	4.13	2.05
AFUA_5G03490	Nucleoside diphosphate kinase	2.17	1.12	4.12	2.04
AFUA_2G06150	Disulfide isomerase, putative	2.34	1.23	3.90	1.96
AFUA_5G00230	Hypothetical protein	4.09	2.03	3.84	1.94
AFUA_6G07290	Endosomal cargo receptor (Erv14), putative	2.63	1.40	3.73	1.90
AFUA_1G16690	MFS transporter, putative	2.06	1.04	3.73	1.90
AFUA_3G06710	Ubiquitin thiolesterase (OtuB1), putative	2.28	1.19	3.61	1.85
AFUA_3G11260	Ubiquitin (UbiC), putative	2.12	1.08	3.57	1.83
AFUA_3G07890	Endo alpha-1,4 polygalactosaminidase, putative	3.47	1.79	3.54	1.82
AFUA_6G04780	Vacuolar protein sorting 55 superfamily	2.43	1.28	3.46	1.79
AFUA_5G11850	Mitochondrial carrier protein (Pet8), putative	2.12	1.08	3.39	1.76
AFUA_5G05450	Ribosomal protein S3Ae cytosolic	2.28	1.19	3.29	1.72
AFUA_6G00680	Hypothetical protein	4.11	2.04	3.26	1.71
AFUA_5G02780	Nicotinamide nucleotide transhydrogenase	2.61	1.38	3.24	1.70
AFUA_1G04040	Ubiquitin (UbiA), putative	2.89	1.53	3.23	1.69
AFUA_1G16030	Conserved hypothetical protein	2.61	1.38	3.22	1.69
AFUA_6G13250	Ribosomal protein L31e	2.16	1.11	3.19	1.68
AFUA_2G05150	Putative cell wall galactomannoprotein Mp2/allergen F17-like	7.91	2.98	3.14	1.65
AFUA_3G13320	40s ribosomal protein S0, putative	2.6	1.38	3.13	1.64
AFUA_6G03830	Ribosomal protein L14	3.04	1.60	3.09	1.63
AFUA_1G10380	Nonribosomal peptide synthase (NRPS)	3.63	1.86	3.09	1.63
AFUA_1G04660	Ribosomal L15	2.08	1.06	3.06	1.61
AFUA_4G01290	Endo-chitosanase, pseudogene	15.82	3.98	3.01	1.59
AFUA_3G10730	Ribosomal protein S7e	2.16	1.11	3.00	1.59
AFUA_5G09400	Carbonyl reductase, putative	3.64	1.86	3.00	1.59
AFUA_4G04070	Conserved hypothetical protein	2.06	1.04	2.96	1.57
AFUA_2G14490	Endoglucanase, putative	32.7	5.03	2.90	1.54
AFUA_4G13080	Monosaccharide transporter	3.91	1.97	2.88	1.53
AFUA_3G05600	Ribosomal protein L27a	2.14	1.10	2.88	1.53
AFUA_7G01590	Cystathionine gamma-synthase	2.11	1.08	2.81	1.49
AFUA_7G05300	Hypothetical protein	3.1	1.63	2.78	1.47
AFUA_1G09510	GPI anchored protein, putative	3.55	1.83	2.76	1.46
AFUA_4G00800	MFS monosaccharide transporter, putative	7.39	2.89	2.73	1.45
AFUA_5G08030	Cellulase CelA, putative	2.23	1.16	2.69	1.43
AFUA_7G04290	Amino acid permease (Gap1), putative	3.43	1.78	2.68	1.42
AFUA_5G03010	Conserved hypothetical protein	6.64	2.73	2.66	1.41
AFUA_1G11670	Small nuclear ribonucleoprotein (LSM8)	2.21	1.14	2.53	1.34
AFUA_4G03760	Glycine dehydrogenase	2.79	1.48	2.51	1.33
AFUA_1G11130	60s ribosomal protein yl16a	2.37	1.24	2.48	1.31
AFUA_1G09530	Conserved hypothetical protein	2.24	1.16	2.48	1.31
AFUA_3G06640	40s ribosomal protein s27 type	2.32	1.21	2.47	1.31
AFUA_7G05290	Cytosolic small ribosomal subunit S15, putative	2.31	1.21	2.43	1.28
AFUA_4G07300	Hypothetical protein	4.85	2.28	2.42	1.27
AFUA_2G02150	Ribosomal protein S10	2.32	1.21	2.40	1.27
AFUA_1G12350	Extracellular fruiting body protein, putative	8.63	3.11	2.39	1.26
AFUA_3G06840	Cytosolic small ribosomal subunit S4, putative	2.41	1.27	2.30	1.20
AFUA_2G02990	MYB DNA-binding domain protein	2.43	1.28	2.27	1.18
AFUA_5G02950	Conserved hypothetical protein	2.7	1.43	2.24	1.16
AFUA_7G08240	Hypothetical protein	2.03	1.02	2.17	1.12
AFUA_1G08880	Heavy metal ion transporter, putative	2.35	1.23	2.10	1.07
AFUA_6G12820	MAP kinase (FUS3/KSS1), putative	3.84	1.94	2.06	1.04
AFUA_7G02570	Heterokaryon incompatibility protein (Het-C)	3.96	1.99	2.05	1.04
AFUA_1G06770	Ribosomal protein S26e	2.98	1.58	2.03	1.02
AFUA_6G06500	Actin-related protein 2/3 complex subunit 1A	2.13	1.09	2.02	1.02
AFUA_5G09750	Nucleoside transporter, putative	2.94	1.56	2.01	1.01
AFUA_1G09690	tRNA liGase	0.37	−1.43	0.49	−1.03
AFUA_7G04010	Conserved hypothetical protein	0.44	−1.18	0.49	−1.03
AFUA_5G01030	Glyceraldehyde 3-phosphate dehydrogenase	0.03	−5.06	0.49	−1.04
AFUA_2G09510	Hypothetical protein	0.17	−2.56	0.48	−1.05
AFUA_4G11560	Vacuolar protein sorting-associated protein vps13	0.45	−1.15	0.48	−1.05
AFUA_2G11460	C6 finger domain protein, putative	0.32	−1.64	0.46	−1.12
AFUA_6G02280	Allergen Asp F3	0.32	−1.64	0.45	−1.16
AFUA_2G15430	L-xylulose reductase	0.13	−2.94	0.44	−1.19
AFUA_1G01600	Deoxyribodipyrimidine photolyase	0.2	−2.32	0.42	−1.25
AFUA_7G01800	AT DNA binding protein, putative	0.27	−1.89	0.41	−1.28
AFUA_3G00370	D-fructose 6-phosphate phosphoketolase	0.08	−3.64	0.40	−1.32
AFUA_3G03400	Siderophore biosynthesis protein, putative	0.46	−1.12	0.39	−1.35
AFUA_2G04190	Hypothetical protein	0.28	−1.84	0.37	−1.44
AFUA_2G06270	Hypothetical protein	0.38	−1.40	0.35	−1.53
AFUA_2G13030	Phenylalanyl-tRNA synthetase alpha subunit (PodG)	0.36	−1.47	0.32	−1.63
AFUA_5G11190	Hypothetical protein	0.14	−2.84	0.32	−1.64
AFUA_5G10660	Pentatricopeptide repeat protein	0.34	−1.56	0.32	−1.66
AFUA_5G08200	Hypothetical protein	0.25	−2.00	0.31	−1.70
AFUA_2G11840	Transcriptional corepressor (Cyc8), putative	0.42	−1.25	0.30	−1.75
AFUA_3G14590	Copper amine oxidase	0.28	−1.84	0.28	−1.85
AFUA_2G09350	Endo-beta-1,6-glucanase, putative	0.37	−1.43	0.27	−1.88
AFUA_2G05450	64 kDa mitochondrial NADH dehydrogenase	0.41	−1.29	0.27	−1.92
AFUA_4G08170	Succinate-semialdehyde dehydrogenase	0.43	−1.22	0.25	−1.98
AFUA_5G14680	Hypothetical protein	0.18	−2.47	0.25	−2.02
AFUA_8G05380	Hypothetical protein	0.48	−1.06	0.22	−2.21
AFUA_2G01140	GPI anchored protein, putative	0.31	−1.69	0.21	−2.23
AFUA_2G07680	L-ornithine N5-oxygenase	0.46	−1.12	0.21	−2.25
AFUA_3G05780	GATA transcription factor (LreA), putative	0.13	−2.94	0.20	−2.29
AFUA_2G16520	Phospholipase D (PLD), putative	0.42	−1.25	0.20	−2.30
AFUA_6G04920	NAD-dependent formate dehydrogenase	0.27	−1.89	0.17	−2.57
AFUA_8G05600	Hypothetical protein	0.07	−3.84	0.16	−2.64
AFUA_1G13800	mfs-multidrug-resistance transporter	0.28	−1.84	0.16	−2.65
AFUA_5G06240	Alcohol dehydrogenase. putative	0.09	−3.47	0.15	−2.72
AFUA_8G05580	Coenzyme A transferase PsecoA	0.19	−2.40	0.15	−2.76
AFUA_4G08960	GPI anchored protein, putative	0.19	−2.40	0.14	−2.86
AFUA_2G13830	Conserved hypothetical protein	0.41	−1.29	0.13	−2.95
AFUA_5G07590	Hypothetical protein	0.32	−1.64	0.13	−2.96
AFUA_7G08280	Hypothetical protein	0.35	−1.51	0.12	−3.02
AFUA_2G09220	Hypothetical protein	0.31	−1.69	0.12	−3.04
AFUA_1G03610	Hypothetical protein	0.11	−3.18	0.12	−3.05
AFUA_1G12250	Mitochondrial hypoxia responsive protein	0.16	−2.64	0.12	−3.07
AFUA_1G10610	Hypothetical protein	0.27	−1.89	0.11	−3.16
AFUA_6G13380	Hypothetical protein	0.4	−1.32	0.11	−3.24
AFUA_3G05760	C6 transcription factor (Fcr1), putative	0.09	−3.47	0.10	−3.34
AFUA_4G11720	Phosphatidyl synthase	0.12	−3.06	0.10	−3.39
AFUA_1G12840	Nitrite reductase	0.2	−2.32	0.09	−3.49
AFUA_5G12530	Conserved hypothetical protein	0.42	−1.25	0.08	−3.68
AFUA_4G03460	HLH DNA binding domain protein, putative	0.13	−2.94	0.08	−3.72
AFUA_3G11070	Pyruvate decarboxylase PdcA, putative	0.1	−3.32	0.07	−3.80
AFUA_3G10750	Acetate kinase, putative	0.34	−1.56	0.06	−4.04
AFUA_4G03410	Flavohemoprotein	0.07	−3.84	0.05	−4.32
AFUA_1G12830	Nitrate reductase NiaD	0.14	−2.84	0.05	−4.40
AFUA_1G15270	ATP-dependent Clp protease, putative	0.17	−2.56	0.02	−5.44
AFUA_3G14540	30 kDa heat shock protein	0.23	−2.12	0.02	−5.44
AFUA_2G05060	Alternative oxidase	0.03	−5.06	0.02	−5.86
AFUA_5G02700	Multidrug resistant protein	0.07	−3.84	0.01	−6.09

A large proportion of common genes up regulated in the BF are involved in the transcriptional and translational regulation reflecting the establishment of different transcriptional and translational programs between these two growth conditions.

Genes coding for antigenic and allergenic proteins are differentially expressed in the BF. Two of the major allergens of *A. fumigatus*, the ribotoxin Asp F1 and the allergen Asp F7-like (extracellular cellulase CelA) are up regulated in the *A. fumigatus* BF (Madan et al., 1997a,b; Alvarez-Garcia et al., 2010). Among the 81 allergens identified in *A. fumigatus*, 39 genes were shown to be up regulated under BF conditions by using RNA-sequencing (Mari and Scala, 2006). Noteworthy, the secreted galactomannoprotein Afmp1p and the mannoprotein Afmp2p are up regulated in the BF (Woo et al., 2002; Chong et al., 2004). Afmp1p and Afmp2p are specific to *A. fumigatus* and are not found in other *Aspergillus* species. A clinical evaluation of sera from invasive aspergillosis patients has revealed that they contained circulating Afmp1p proteins as well as antibodies directed against both Afmp1p and Afmp2p proteins. A dual detection system was suggested for the diagnosis of aspergillosis based on the presence of circulating Afmp1 antigen and antibodies against Afmp2p. An overexpression of antigenic molecule does not occur in all cases, e.g., the allergen thioredoxin peroxidase AspF3 is down regulated in the BF (Kniemeyer et al., 2009). The occurrence of a higher production of allergens/antigens in the BF condition is in agreement with the initial observations that growth of the fungus in an infected lung is similar to the *in vitro* BF growth.

The *rodB* gene belonging to the hydrophobins family is also highly up regulated in the BF. *A. fumigatus* has at least six genes that code for hydrophobins, but only *rodA* and *rodB* have been studied for virulence implications (Paris et al., 2003). The *rodA* gene encodes a small hydrophobic cysteine-rich polypeptide present on the surface of the conidia and the deletion mutant displays a conidial cell wall without rodlet layer allowing a better recognition to alveolar macrophages. The *rodA* mutant produced smaller lung lesions and weaker inflammatory response than the reference wild-type strain in a murine model of invasive aspergillosis. However, although the *rodB* gene is highly expressed in the BF, the *rodB* deletion mutant did not show any obvious morphological phenotypes. The role of this hydrophobin in mycelial growth remains obscure.

The gene coding for the putative *O*-methyltransferase CalO6 is one of the most up regulated gene in the BF found in both analyses. This gene belongs to a secondary metabolism supercluster responsible for the biosynthesis of fumitremorgin, pseurotin A, and an unknown secondary metabolite (Khaldi et al., 2010). Among this supercluster composed of 44 genes, 3 genes were found to be up regulated in the microarray data set in comparison to 32 up regulated genes identified by RNA-sequencing. Fumitremorgin was shown to be an inhibitor of chemotherapy-resistant breast cancer cells and conferred sensitivity to anticancer drugs (Grundmann et al., 2008). In spite of these interesting biological characteristics, the potential role of fumitremorgins in *Aspergillus* pathogenesis has not been elucidated yet. The role of the pseurotin A toxin in the pathogenesis of *A. fumigatus* is also poorly understood (Ishikawa et al., 2009; Vodisch et al., 2011). The pseurotin A toxin was shown to be produced under hypoxic conditions and showed a slight cytotoxicity against lung fibroblasts and the capacity to inhibit IgE production (Ishikawa et al., 2009). Most of the studies on *Aspergillus fumigatus* mycotoxins dealt with gliotoxin. The corresponding gene cluster of gliotoxin is up regulated in the BF (Bruns et al., 2010; Speth et al., 2011; Scharf et al., 2012). Even though their role in fungal pathogenicity was suggested by these studies, their role during infection has not been experimentally assessed using pure substance.

**Figure 3:**
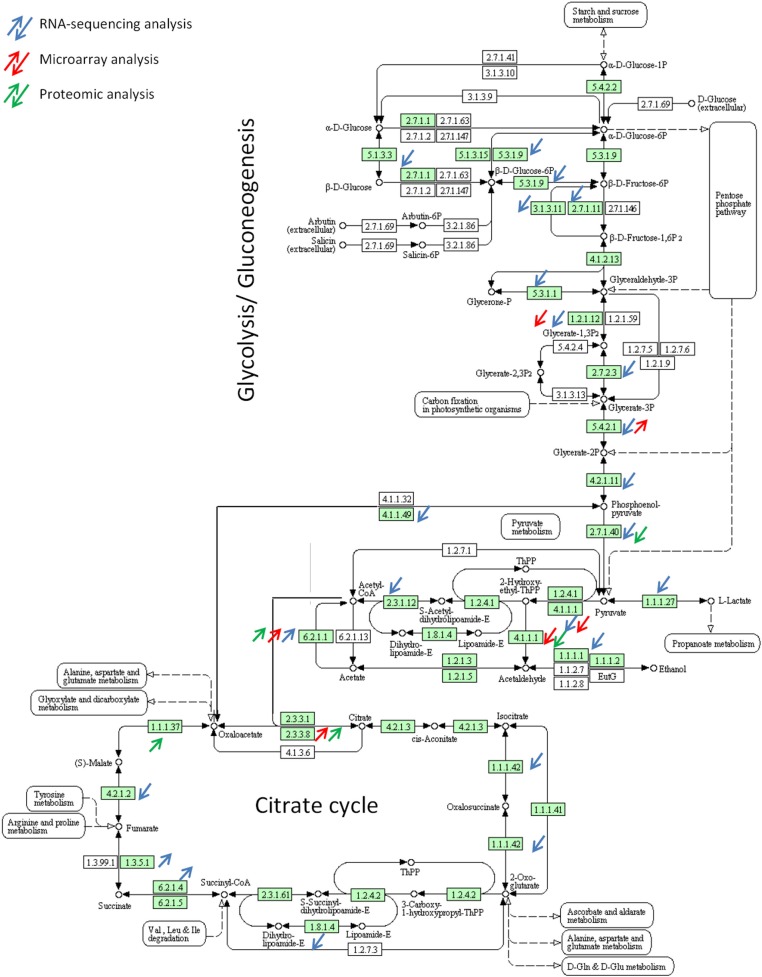
**Differential expression of genes involved in glycolysis pathway and TCA cycle during biofilm growth**.

RNA-sequencing as compared to microarrays provides clear evidence that entire pathways are differentially expressed. For example, the glycolysis pathway responsible for the conversion of glucose to pyruvate was shown to be down regulated in the *A. fumigatus* BF in both transcriptomic methods. Whereas microarrays allowed the identification of only 5 down regulated genes of the glycolysis, the RNA-sequencing highlighted 17 down regulated genes out of 28 genes constituting the glycolysis pathway (Figure 3). Genes encoding enzymes of the tricarboxylic-acid cycle are also differentially expressed as revealed by both transcriptomic methods. Genes encoding enzymes responsible of the conversion of citrate to succinyl-CoA, the oxidative branch of the TCA cycle, were shown to be down regulated in RNA-sequencing whereas enzymes participating in the conversion of succinyl-CoA to oxaloacetate were shown to be up regulated. In line with this, the isocitrate lyase, which is involved in the conversion of isocitrate to glyoxylate and succinate was shown to be up regulated in both analyses. These results reflect that the fungus may not acquire energy by fermentation but by metabolizing acetyl-CoA using the glyoxylate cycle under BF conditions. NADH formed by this cycle can enter then in the respiratory chain pathway. Genes belonging to the mitochondrial complexes II, III, and V, controlling oxidative phosphorylation, were shown to be up regulated in the BF in the RNA-sequencing analysis. In *Candida albicans*, levels of isocitrate lyase and malate synthase are greatly increased upon contact with its human host and interestingly, isocitrate lyase has been shown to be key virulence factor (Lorenz and Fink, 2001). In contrast, isocitrate lyase of *A. fumigatus* is not essential for the development of invasive aspergillosis in a murine model (Schobel et al., 2007).

One hundred and forty transporter genes were up regulated in the BF based on RNA-sequencing analysis. In comparison, microarrays revealed only the up regulation of only 5 MFS and 3 ABC transporters. The *Mdr4* transporter was shown to be up regulated in an *in vivo* BF mouse model during voriconazole treatment (Langfelder et al., 2002; Nascimento et al., 2003; Rajendran et al., 2011). The ABC transporters *Mdr1, Mdr2*, and *Mdr4* which are overexpressed in itraconazole-resistant mutants induced *in vitro* are also up regulated in our BF condition in the RNA-sequencing analysis (Nascimento et al., 2003). Thus, the up regulation of these efflux pumps in *A. fumigatus* could lead to azole resistance in BF grown *A. fumigatus* cultures. A recent study showed that the *A. fumigatus* BF sensitivity to voriconazole was increased in presence of an efflux pump inhibitor reflecting the importance of the transport activity in the BF to counteract the action of inhibitors in association with the 14-α-demethylase *Cyp51A* (Rajendran et al., 2011).

## Proteomics analysis

**Figure 4:**
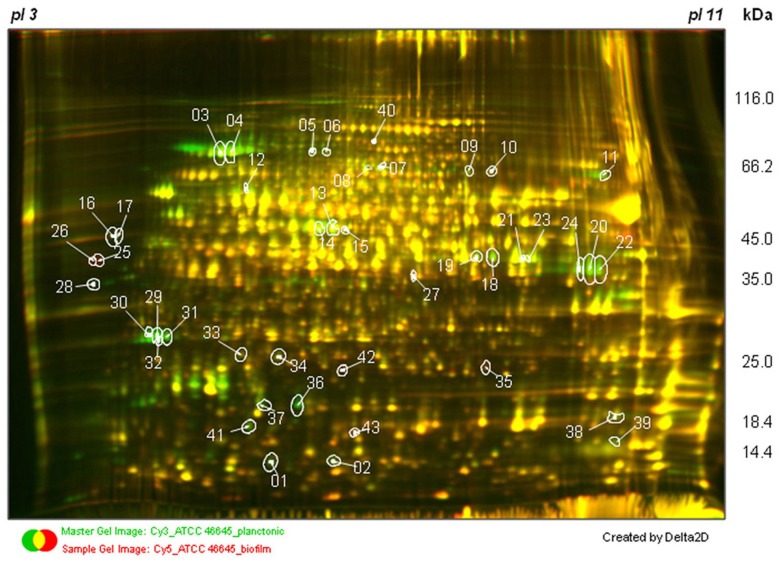
**2D electrophoretic separation of protein extracts of *A. fumigatus* grown under submerged (PL) and biofilm (BF) culture conditions.** In total, 43 different protein spots of *A. fumigatus* changed significantly their abundance within 16 h of growth (protein spots are labeled with spot numbers as indicated in Table 5). *A. fumigatus* proteins were labeled with the CyDye DIGE Fluor minimal dye labeling kit. Subsequently, proteins were separated by 2D gel electrophoresis using immobilized pH gradient strips with a pH range of 3–11 NL in the first dimension. For the separation of proteins in the second dimension, SDS-polyacrylamide gradients gels (11–16%) were used. Differentially regulated proteins were identified by MALDI-TOF/TOF analysis. A three color overlaid gel image is shown. Samples were labeled as follows: ATCC 46645-planctonic culture control sample (Cy3), ATCC 46645-biofilm culture sample (Cy5), and internal standard (Cy2).

Large-scale analysis of the proteome is also important for a better understanding of the cellular, metabolic, and regulatory networks in the cell. Proteomic analysis offers the advantage to visualize the final product of the gene transcription. This methodology has still a bias against low-abundance and membrane proteins. However, targeted proteomic approaches based on LC-MS/MS techniques, such as selected reaction monitoring (SRM), have the potenial to detect proteins with low copy numbers (Picotti et al., 2009). In *A. fumigatus*, around 650 proteins have so far been identified by 2D-gel electrophoresis for a genome that has ~10,000 genes (Teutschbein et al., 2010). The proteomic analysis of the BF condition after 16 h growth as compared to submerged condition was performed as described by Bruns et al. (2010), with slight modifications. 2D-gel images were analyzed by using Delta 2D 4.3 (Decodon, Germany). Analysis of the 2-D gel patterns obtained revealed that 43 spots showed significant changes in abundance between the BF and planktonic cultures (Figure 4). Among them, 25 different proteins were identified by MALDI-TOF/TOF-analyses (Table 5). Three proteins were up and 22 were down regulated under BF conditions.

**Table 5:** **List of differentially expressed proteins obtained by using proteomic analysis**.

	**Accession number**	**Spot number**^**a**^	**Putative function**	**Proteomic ratio of biofilm/planctonic conditions**^**b**^	**RNA-seq ratio of biofilm/planctonic conditions**^**c**^
1	AFUA_1G05340	38	40s ribosomal protein S19	0.43	4.21
2	AFUA_1G07480	15	Coproporphyrinogen III oxidase	0.39	
3	AFUA_1G12890	20	Probable 60s ribosomal protein l5	0.40	
		22		0.40	
4	AFUA_1G14120	11	Nuclear segregation protein (Bfr1)	0.47	
5	AFUA_1G16840	32	TCTP family protein	0.43	2.96
6	AFUA_2G04060	15	NADH:flavin Oxidoreductase/NADH oxidase family protein	0.39	0.14
7	AFUA_2G07420	6	Actin-bundling protein Sac6	0.39	
8	AFUA_2G11010	9	Dihydroorotate reductase PyrE	0.15	
		10		0.37	
9	AFUA_2G11150	12	Secretory pathway gdp dissociation inhibitor	0.49	
10	AFUA_3G00590	41	Asp-hemolysin	0.36	
11	AFUA_3G08420	34	Cystathionine beta-synthase (beta-thionase)	0.45	
12	AFUA_3G12300	39	60s ribosomal protein L22	0.37	5.60
13	AFUA_4G03410	13	Flavohemoprotein	0.28	0.05
		14		0.32	
14	AFUA_4G08240	19	Zinc-containing alcohol dehydrogenase	0.47	0.05
15	AFUA_4G11080	40	Acetyl-coenzyme A synthetase FacA	2.40	0.29
16	AFUA_5G02370	6	Vacuolar ATP synthase catalytic subunit A	0.39	
17	AFUA_5G06240	18	Alcohol dehydrogenase, putative	0.24	0.15
		23		0.35	
18	AFUA_5G09910	33	Nitroreductase family protein	0.38	0.12
19	AFUA_5G14680	29	Conserved hypothetical protein	0.20	0.25
		30		0.32	
		31		0.14	
20	AFUA_6G02420	43	Ubiquitin conjugating enzyme (UbcM)	2.01	4.77
21	AFUA_6G02750	36	Nascent polypeptide-associated complex (NAC) subunit	0.42	2.09
22	AFUA_6G05210	27	Malate dehydrogenase, NAD-dependent	2.12	
23	AFUA_6G07430	7	Pyruvate kinase	0.45	0.43
24	AFUA_7G01010	19	Alcohol dehydrogenase	0.47	83.45
25	AFUA_8G03930	3	Hsp70 chaperone (HscA)	0.46	0.46
		4		0.49	
		5		0.45	

a Spot number in Figure 4.

b Average ratios compared under biofilm and planctonic growth conditions were extracted from statistical analysis of DIGE gels by the Delta2D 4.3 (Decodon) software program. “>2” a consistent increase of greater than twofold. “<2” a consistent decrease of more than twofold.

c The transcriptional changes determined by RNA-seq are aligned.

### Proteomic vs. RNA-seq data

The comparison of the transcriptomic and proteomic data has revealed that 16 genes corresponding to differentially regulated proteins were retrieved in the RNA-sequencing data vs. only 5 genes for microarrays. Only 8 of the 22 down regulated proteins and corresponding mRNA were found to be down regulated (cutoff <0.5) with a correlation of *p* = 0.43 (Pearson correlation) and one protein and its corresponding mRNA was up regulated. These results stressed the difficulties in correlating transcriptome and proteome data. Several reasons may explain the low number of differentially expressed proteins and the low degree of correlation between transcriptomic and proteomic analyses (Nie et al., 2007; Sukardi et al., 2010). For technical reasons, the current two-dimensional gel-based analyses focus mainly on the cytoplasmic subset of the cell proteome due to the impossibility to date to extract most membrane or hydrophobic proteins. Proteins are then separated according to their isoelectric point and molecular mass. So proteins with an extreme isoelectric point or molecular mass are not amenable to 2D-gel electrophoresis. A sufficient amount of protein present in one spot is also crucial for the unambiguous identification of the protein by MALDI-TOF/TOF-analyses. Conversely, RNA-sequencing allows the identification of thousand mRNAs differentially regulated between two conditions. However, the transcript levels detected in mRNA profiling do not reflect all the regulatory processes in the cell, such as post-transcriptional-processes occurring before translation, the half-lives of mRNAs and proteins and the post-translational regulation on the protein level as the quality control of proteins and the degradation in the proteasome. Conversely to RNA-sequencing, the proteomic analysis highlights fewer regulated proteins but assured their real up regulation or down regulation in the cell. Thus, even if a limited number of proteins were identified by proteomic analysis, some of them could confirm the up regulation of pathways or genes in the BF at the protein level.

Among the proteins identified, proteins involved in the translational regulation and post-translational modifications are found. The data were in agreement with the transcriptomic data and shows that the transcriptional and translational processes involved in the two growth conditions were different.

Similarly, the pyruvate kinase was down regulated whereas the acetyl-CoA synthetase FacA and the malate dehydrogenase were up regulated in the BF. These results confirmed the down regulation of the glycolysis pathway and the up regulation of final steps of the TCA at the protein level.

The Asp-hemolysin protein was down regulated in the BF. Asp-hemolysin was reported to be released into the culture supernatant by *A. fumigatus* during growth in presence of elastin, collagen, and keratin, where it is supposed to exhibit a hemolytic activity (Wartenberg et al., 2011). However, the characterization of the deletion strain Δ*asp*-HS did not revealed significant hemolytic and cytotoxic activity and the impact on pathogenicity and the biological role of the Asp-HS protein is still poorly understood.

All proteome data (gel images, spot information) were imported into our in-house data ware-house Omnifung http://www.omnifung.hki-jena.de and are publicly accessible.

## Conclusions

In recent years, many high-throughput technologies have been developed to decipher various aspects of cellular processes, including the transcriptome, epigenome, proteome, metabolome, or interactome. The capacity to perform “omics” analyses at several different levels, such as transcriptomic, proteomic, or metabolomics, and their comparison and integration of information offers an exciting potential to answer many questions asked by a biological study. However, even if the utilization of different “omics” methods can be complementary, the combination of the different data obtained remains a challenge. Among the three “omics” methods used to identify the specific signature of the *A. fumigatus* BF, the RNA-sequencing has exceeded microarrays and is the most powerful analysis giving precise information on the expression of the entire genes of the genome in a biological sample with a few degree of variability. RNA-sequencing has allowed the identification of up regulated genes involved in transport, secondary metabolism, antigenic and allergenic molecules during BF growth. Data obtained have reflected the metabolic reorganization occurring in the BF. Thus, RNA-sequencing allows the identification of the genes differentially expressed between two biological conditions, but it also provides information concerning sequence variations such as alternative splicing events, gene fusion detection, and small RNA characterization at single-nucleotide resolution (Morozova et al., 2009). In contrast, proteomic analysis allows the identification of proteins, the final product of the gene expression, but the information collected is limited due to the high dynamic range of protein concentration within a cell and the difficulties in analyzing membrane proteins. However, the tremendous progress in LC-MS/MS-based proteomics, which has recently been made, opens up the possibility to detect and quantify also low abundant, highly glycosylated, and hydrophobic proteins including membrane proteins (Savas et al., 2011). To date even though these “omics” technologies are very appealing, the data obtained so far have not yet been able to solve the identification of virulence factors in *A. fumigatus*. Due to the opportunistic pathogenicity of the species, the identification of the essential metabolic pathways under *in vivo* conditions may be a better option than the search for specific virulence factors. In this option, “omics” technologies have a great future in the field of human-pathogenic fungi.

### Conflict of interest statement

The authors declare that the research was conducted in the absence of any commercial or financial relationships that could be construed as a potential conflict of interest.
